# Singing as a rehabilitation method from the viewpoint of Avicenna (980–1037 AD)

**DOI:** 10.1038/s41533-017-0021-2

**Published:** 2017-03-07

**Authors:** Narjes Gorji, Reihaneh Moeini

**Affiliations:** Department of Traditional Iranian Medicine, School of Traditional Iranian Medicine, Traditional Medicine and History of Medical Sciences Research Center, Babol University of Medical Sciences, Babol, Iran

We read with great interest your published article entitled “singing for Lung Health: a systematic review of the literature and consensus statement” that explained the effect of singing on lung function.^1^ In the background, authors pointed to singing therapy as a novel approach for respiratory disease treatment;^1^ but it must be said there is already some evidence of this discovery found many centuries ago.^2^

In reviewing the scientific achievements, experiences and knowledge of predecessors should not be ignored. Avicenna as the most talented scholar of Persian medicine (980–1037 AD)^3^ more than a thousand years ago, recommended signing, for improving the function of respiratory system, increasing lung capacity, strengthening chest muscle, and clearing the mucus of the lung.^2^

In the second section of the first book of *the canon of medicine*, he explained singing as an especial kind of exercise for eye, mouth, tongue, pharynx, and lung that could be helpful for health maintenance and disease treatment of these organs. He wrote: “singing must start with a low voice intensity then it should rise up gradually and then stay in high for a while. This has an obvious and considerable therapeutic benefit”.^2^

He described clearly the effects of singing on improvement of some lung diseases like asthma and dyspnea. Moreover, he believed signing, besides the breathing exercise, can improve cerebral circulation and mental function. He also emphasized that signing has an amazing effect on psychological rehabilitation especially for nervous patient and it also improves blood circulation and skin lightening, but he does not prescribe loud singing for a long period.^2^

According to several studies which have confirmed the views of Avicenna,^4, 5^ it seems that more attention should be paid to his ideas in research in various fields of medicine including lung disease.
